# Restoration of Elbow Flexion in Patients With Complete Traumatic and Obstetric Brachial Plexus Injury After Functional Free Gracilis Muscle Transfer: Our Experience and Management

**Published:** 2017-11-21

**Authors:** Rahul K. Nath, Sean G. Boutros, Chandra Somasundaram

**Affiliations:** ^a^Texas Nerve and Paralysis Institute, Houston; ^b^Houston Plastic Craniofacial and Sinus Surgery, Houston

**Keywords:** brachial plexus injury, voluntary elbow flexion, biceps muscle function, nerve avulsion, gracilis muscle transfer

## Abstract

**Background:** Functional free gracilis muscle transfer is an operative procedure for elbow reconstruction in patients with complete brachial plexus nerve and avulsion injuries and in delayed or prolonged nerve denervation, as well as in patients with inadequate upper extremity function after primary nerve reconstruction. **Methods:** We retrospectively reviewed our patient records and identified 24 patients with complete brachial plexus nerve injury (13 obstetric, 11 males and 2 females; 11 traumatic, 9 males and 2 females) whose affected arm and shoulder were totally paralyzed and their voluntary elbow flexion or the biceps function was poor preoperatively (mean M0-1/5 in MRC grade). These patients had undergone the functional free gracilis muscle transfer procedure at our clinic since 2005. **Results:** Ninety-two percent of all patients showed recovery and improvement. Successful free gracilis muscle transfer is defined as antigravity biceps muscle strength of M3-4/5 and higher, which was observed in 16 (8 obstetric and 8 traumatic) of our 24 patients (67%) in this study at least 1 year after functional free gracilis muscle transfer. This is statistically significant (*P* < .000001) in comparison with their mean preoperative score (M0-1/5). There was no improvement in motor level of the biceps muscle (M0/5) in 2 patients (1 from each group). The donor site of these 24 patients showed no deficit in motor and sensory functions. **Conclusions:** Taken together, a significant number (92%) of patients in both obstetric and traumatic brachial plexus injury groups had recovery and improvement and most of these patients (64%) achieved antigravity and elbow flexion at least 1 year after free gracilis muscle transfer at our clinic.

Functional free gracilis muscle transfer (FFGMT) is an operative procedure for restoration of elbow flexion in patients with complete brachial plexus nerve injuries and in delayed or prolonged nerve denervation, as well as in patients with inadequate upper extremity function after primary nerve reconstruction.^1^^-^9

In a total or complete brachial plexus injury (CBPI), the entire plexus is injured and that is more devastating. Twenty percent of patients with obstetric brachial plexus injury (OBPI) are CBPI, which severely compromises the patient's overall upper extremity functions and growth.^10^^,^^11^ Traumatic brachial plexus injury (TBPI) is also a severe and devastating condition observed in up to 4.2% of multitrauma victims.^12^ The management of complete or avulsion (preganglionic lesion) injuries is challenging using mainly nerve transfer or graft procedures in both OBPI and TBPI patients due to its complexity of the injury.

Functional free muscle transfer (FFMT) with nerve transfer has been commonly adopted for gaining elbow flexion in these patients.^1^^-^9 Limited donor nerve for nerve transfer and long distances to the target muscle are the main obstacles in these patients. Therefore, the FFMT is the only option to improve their limb function. The functioning gracilis muscle is used as the functional deficit after gracilis harvest is negligible.^1^

Here, we report the outcomes, in particular, the biceps function/elbow flexion after FFGMT in 24 patients with complete brachial plexus nerve injury (13 obstetric and 11 traumatic) who lost their biceps strength and function.

## PATIENTS AND METHODS

### Inclusion criteria

Both OBPI and TBPI patients, who had no visible biceps function and no elbow flexion, were included.

We retrospectively reviewed our patient records with brachial plexus nerve injury and identified 24 severely paralyzed patients (13 obstetric, 11 males and 2 females; 11 traumatic, 9 males and 2 females) who had no elbow flexion and had undergone FFGMT at our clinic since 2005. The median (18), radial (5), and ulnar (1) nerves reinnervated the transferred gracilis muscle in our patients with brachial plexus injury (BPI) in the present study.

Results were assessed by the MRC grading system. Mean age at the time of surgery was 10 years (range, 5.4-14.2 years) in obstetric patients and 27 years (range, 8-50 years) in traumatic patients. Voluntary elbow flexion or the biceps function was very poor before FFGMT (mean = M0-1/5 in MRC grade) in most of our patients in this study.

Written informed consent was obtained from all patients for publication and accompanying images. A copy of the written consent is available for review on request. This was a retrospective study of patient charts, which exempted it from the need for institutional review board approval in the United States. Patients were treated ethically in compliance with the Helsinki declaration. Documented informed consent was obtained for all patients.

## RESULTS

Taken together, a significant number of patients (92%) in both OBPI and TBPI groups had recovery and improvement (Table 1).

**Figure 1:**
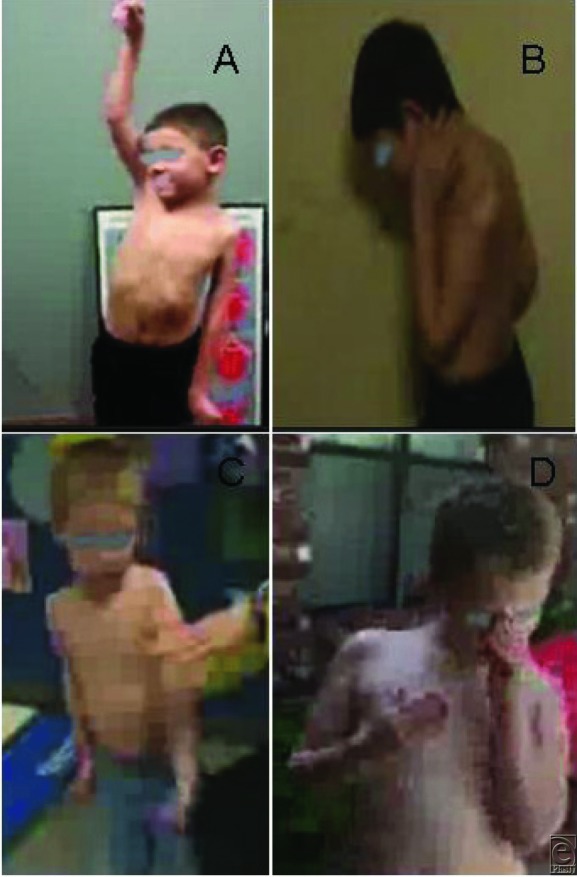
(a) A 7-year-old TBPI (MVA) patient with complete loss of left shoulder movements. (b) The same TBPI patient with restored elbow flexion 1 year after FFGMT. (c) A 6-year-old OBPI patient with complete loss of upper extremity movements (left). (d) The same OBPI patient with fully restored elbow flexion 4 years after FFGMT. TBPI indicates traumatic brachial plexus injury; MVA, motor vehicle accident; FFGMT, functional free gracilis muscle transfer; and OBPI, obstetric brachial plexus injury.

Successful FFGMT is defined as antigravity biceps muscle strength of M3-4/5 and higher, which was observed in 16 (8 obstetric and 8 traumatic) of our 24 patients (67%) in this study at least 1 year after FFGMT (Table 1 and Figure 1). This is statistically highly significant (*P* < .000001) in comparison with their mean preoperative score (M0-1/5). There were some recovery and improvement (M1-2/5) in 6 patients (4 from OBPI and 2 from TBPI), but they did not achieve antigravity. There was no improvement in motor level of the biceps muscle (M0/5) in 2 patients (1 from each group).

The donor gracilis muscle of these 24 patients showed no deficit in motor and sensory functions.

## DISCUSSION

Several authors have described a number of functional muscle transfer surgeries involving latissimus dorsi (LD)^13^^-^18 trapezius transfer,^18^^-^20 pectoralis major,^21^^,^^22^ rectus femoris muscle,^23^ and gracilis^1^^-^9 to restore elbow flexion and hand and shoulder functions. These authors have reported a range of outcomes in patients with complete or preganglionic severe BPI. For example, Kawamura et al^24^ reported that 50% of patients did not achieve sufficient elbow flexion after initial LD transfer in a series of 10 patients. The muscle was deemed too long and had to shorten at the distal end of the transfer to achieve better outcomes in this series.^24^ LD transfer in BPI patients is mainly used to restore external rotation at the shoulder.^25^ Maldonado et al^26^ showed 67.7% success in their TBPI patients, achieving elbow flexion after the FFGMT procedure. Gardiner and Nanchahal^27^ found 91% to 99% success using FFGMT. Yet, other investigators^28^ reported an overall failure rate of 15.4%.

Hattori et al^29^ studied comparison between spinal accessory (SAN) and intercostal nerve (ICN) reinnervation and showed that the contraction rate was significantly higher among the transferred muscles reinnervated by the SAN than those by the ICN. We have not found such significant differences in the outcome based on the nerves used in FFGMT in our study patients. We have used a part of the transplanted vascularized median or radial or ulnar nerve as a motor source of a free muscle graft. Chung et al^30^ achieved 78% success using the gracilis muscle in 23 of their patients following transfer of 3 ICNs. These authors also found a greater increase in elbow flexion when ICNs were transferred to innervate the gracilis flap than ulnar fascicles.

## COMPLICATIONS OF FFGMT

Hattori et al^31^ detected obturator nerve injury associated with femur fracture fixation during gracilis muscle harvesting for FFGMT. The most common late complication reported was fracture of the clavicle (5.4%).^28^ One of our patients has lost some strength due to tendon lengthening, although gained antigravity function (biceps M3 and above) after FFGMT. This patient was recommended for FFGMT tightening with longer (6-month) immobilization.

## CONCLUSIONS

Taken together, a significant number (92%) of patients in both OBPI and TBPI groups had recovery and improvement and most of these patients achieved antigravity and elbow flexion at least 1 year after free gracilis muscle transfer at our clinic.

## AUTHOR CONTRIBUTIONS

R.K.N. conceived of the study, R.K.N. and S.G.B. performed all the surgeries and revised the manuscript. C.S. participated in the design of the study, gathered data, performed the statistical analysis, and drafted the manuscript. All authors read and approved the final manuscript.

## Figures and Tables

**Table 1:** Biceps strength and elbow flexion in patients with severe brachial plexus injury after free functional gracilis muscle transfer*

Patient #	Cause of injury	Previous surgeries	Gender and age at surgery	Time from injury to surgery, y	Nerve used in surgery	Follow-up, mo	Preoperative (M)	Postoperative (M)	Surgical outcomes
1	OBPI	None	M/7.6	7.6	Median	18	0	3	Achieved AG, but not full extension
2	OBPI	NT, TT, FO, and HO	M/11.3	11	Median	12	0	2	No AG, but some improvement
3	OBPI	Steindler flexorplasty, WTT, MQ, osteotomy of the wrist	M/10.7	10.7	Median	44	1	3	Achieved AG
4	OBPI	NT	M/5.4	5.4	Radial	38	0	3	Elbow flexion, no ROM
5	OBPI	Primary, MQ, FMT by other surgeon, FO, BTL, TT, WTT	M/8.9	8.9	Median	44	0	0	No change
6	OBPI	Primary, FO and HO	F/10	10	Median	81	2	2	Worsening overall upper extremity functions with age and growth; gracilis tendon tightening scheduled, may benefit from derotational HO in future
7	OBPI	NT, MQ, FO, and HO	F/10.1	10.1	Median	60	1	3	Stable overall function and strength
8	OBPI	Primary, NT	M/18	18	Median	84	0	3	Stable bicep strength
9	OBPI	Primary, NG, MQ, TT, posterior capsulorrhaphy, FO and HO, BTL	M/5.5	5.5	Median	100	1	1+	Elbow flexion contracture is still present, but no management of this is required now, as overall stable and improved shoulder function and position
10	OBPI	Primary, exploratory, capsulodesis, MQ, FO	M/10	10	Median	13	2	4	Outstanding outcome
11	OBPI	Hernia repair, MQ, therapy	M/7.1	7.1	Median	18	0	2	Some improvement
12	OBPI	C7 NT, derotation osteotomy	M/11.5	11	Radial	70	2	3+	Biceps strength is good
13	OBPI	NT, BP exploration	M/14.2	14.2	Median	18	1	3	Weaker biceps in 30 mo evaluation than in 18 mo evaluation, will verify therapy records and follow for further evaluation
14	TBPI	None	F/37.5	2	Median	34	2	2	Weak biceps, will try Botox for triceps; schedule Zancolli lasso for fingers; good strength of FGMT
15	TBPI	None	M/26	4	Median	12	0	1+	Initial recovery of biceps
16	TBPI	Tumor removal, pulley procedure neurolysis, PM TT	M/19	3.5	Median	12	0	3	Achieved AG
17	TBPI	C7 NT	M/21.4		Radial	61	2	4	Great elbow flexion
18	TBPI	Primary, NT, neurolysis	M/29.5	6.3	Ulnar	23	2	3	Achieved AG
19	TBPI	Too many on head and face	M/17	0.8	Median	65	0	4	Much improved biceps strength
20	TBPI	None	M/8	4.4	Median	13	0	3	AG
21	TBPI	C7 NT, therapy	M/25	4.7	Radial	15	0	0	Flicker of gracilis, elbow movement is palpable, but no ROM
22	TBPI	NT	M/35	3	Median	66	2	4	Excellent result in elbow flexion, not gained original strength of heavy lifting for the job
23	TBPI	Nerve procedures	F/30	6	Radial	46	2	3	Ongoing stable and excellent elbow flexion recovery; no finger or wrist movement seen, but there is increased tone in all IP joints of fingers
24	TBPI	Pectoralis transfer by other surgeon	M/50	15	Median	50	0	4+	Ongoing excellent function in bicep strength and elbow flexion
Mean				7.8		41.5	0.8	2.6	
SD				4.4			0.9	1.2	
*P*								.000001	

*OBPI indicates obstetric brachial plexus injury; AG, antigravity; NT, nerve transfer; TT, tendon tightening; NT, nerve transfer; FO, forearm osteotomy; HO, humeral osteotomy; WTT, wrist tendon transfer; MQ, modQuad; ROM, range of motion; FMT, free muscle transfer; BTL, biceps tendon lengthening; TT, triangle tilt; BP, brachial plexus; TBPI, traumatic brachial plexus injury; FGMT, free gracilis muscle transfer; PM, pectoralis major; and IP, interphalangeal.
